# Altered blood gene expression in the obesity-related type 2 diabetes cluster may be causally involved in lipid metabolism: a Mendelian randomisation study

**DOI:** 10.1007/s00125-023-05886-8

**Published:** 2023-02-24

**Authors:** Juliette A. de Klerk, Joline W. J. Beulens, Hailiang Mei, Roel Bijkerk, Anton Jan van Zonneveld, Robert W. Koivula, Petra J. M. Elders, Leen M. ’t Hart, Roderick C. Slieker

**Affiliations:** 1 Department of Cell and Chemical Biology, Leiden University Medical Center, Leiden, the Netherlands; 2 Department of Internal Medicine (Nephrology), Leiden University Medical Center, Leiden, the Netherlands; 3 Amsterdam Public Health Institute, Amsterdam UMC, Amsterdam, the Netherlands; 4 Julius Center for Health Sciences and Primary Care, University Medical Center Utrecht, Utrecht, the Netherlands; 5 Department of Epidemiology and Data Science, Amsterdam UMC, location VUmc, Amsterdam, the Netherlands; 6 Sequencing Analysis Support Core, Leiden University Medical Center, Leiden, the Netherlands; 7 Department of Clinical Sciences, Lund University, Genetic and Molecular Epidemiology, CRC, Skåne University Hospital Malmö, Malmö, Sweden; 8 Department of General Practice and Elderly Care Medicine, Amsterdam Public Health Research Institute, Amsterdam UMC, location VUmc, Amsterdam, the Netherlands; 9 Department of Biomedical Data Sciences, Section Molecular Epidemiology, Leiden University Medical Center, Leiden, the Netherlands

**Keywords:** Clusters, Lipid metabolism, Long non-coding RNA, Obesity, Two-sample Mendelian randomisation, Type 2 diabetes

## Abstract

**Aims/hypothesis:**

The aim of this study was to identify differentially expressed long non-coding RNAs (lncRNAs) and mRNAs in whole blood of people with type 2 diabetes across five different clusters: severe insulin-deficient diabetes (SIDD), severe insulin-resistant diabetes (SIRD), mild obesity-related diabetes (MOD), mild diabetes (MD) and mild diabetes with high HDL-cholesterol (MDH)*.* This was to increase our understanding of different molecular mechanisms underlying the five putative clusters of type 2 diabetes.

**Methods:**

Participants in the Hoorn Diabetes Care System (DCS) cohort were clustered based on age, BMI, HbA_1c_, C-peptide and HDL-cholesterol*.* Whole blood RNA-seq was used to identify differentially expressed lncRNAs and mRNAs in a cluster compared with all others. Differentially expressed genes were validated in the Innovative Medicines Initiative DIabetes REsearCh on patient straTification (IMI DIRECT) study. Expression quantitative trait loci (eQTLs) for differentially expressed RNAs were obtained from a publicly available dataset*.* To estimate the causal effects of RNAs on traits, a two-sample Mendelian randomisation analysis was performed using public genome-wide association study (GWAS) data.

**Results:**

Eleven lncRNAs and 175 mRNAs were differentially expressed in the MOD cluster, the lncRNA *AL354696.2* was upregulated in the SIDD cluster and *GPR15* mRNA was downregulated in the MDH cluster. mRNAs and lncRNAs that were differentially expressed in the MOD cluster were correlated among each other. Six lncRNAs and 120 mRNAs validated in the IMI DIRECT study. Using two-sample Mendelian randomisation, we found 52 mRNAs to have a causal effect on anthropometric traits (*n*=23) and lipid metabolism traits (*n*=10). *GPR146* showed a causal effect on plasma HDL-cholesterol levels (*p* = 2×10^–15^), without evidence for reverse causality.

**Conclusions/interpretation:**

Multiple lncRNAs and mRNAs were found to be differentially expressed among clusters and particularly in the MOD cluster. mRNAs in the MOD cluster showed a possible causal effect on anthropometric traits, lipid metabolism traits and blood cell fractions. Together, our results show that individuals in the MOD cluster show aberrant RNA expression of genes that have a suggested causal role on multiple diabetes-relevant traits.

**Graphical abstract:**

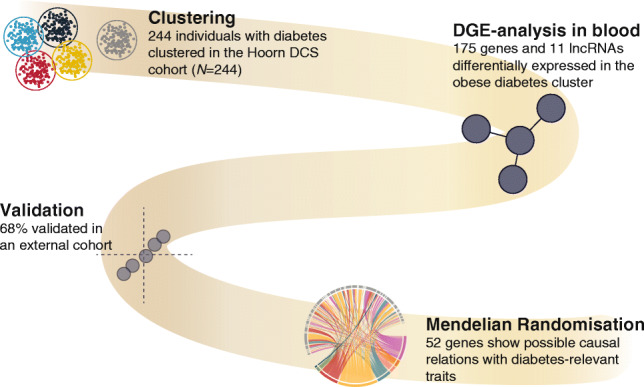

**Supplementary Information:**

The online version of this article (10.1007/s00125-023-05886-8) contains peer-reviewed but unedited supplementary material..

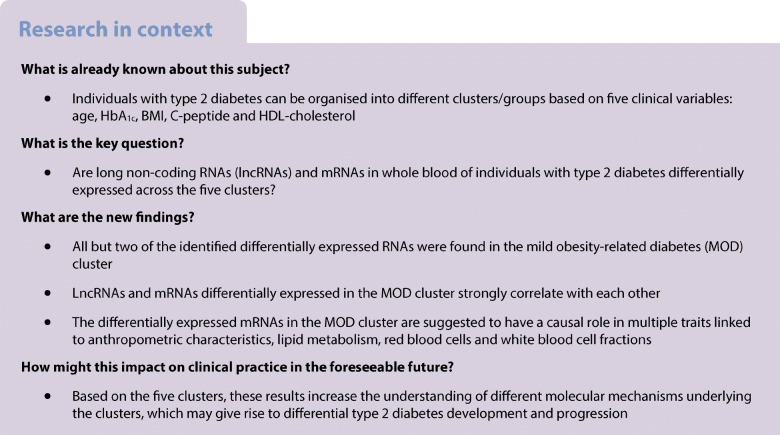



## Introduction

Individuals with diabetes are heterogenous as a group, urging the need for further stratification [1]. It has been shown that individuals with diabetes can be organised into five clusters based on six clinical variables: age, GAD antibodies, BMI, HbA_1c_, insulin resistance (HOMA2-IR) and beta cell function estimates (HOMA2-B) [1].

Among these five clusters was one type 1 diabetes-like cluster (severe autoimmune diabetes, SAID) and four type 2 diabetes clusters, including an insulin-deficient cluster (severe insulin-deficient diabetes, SIDD), an insulin-resistant cluster (severe insulin-resistant diabetes, SIRD), an obesity-related cluster (mild obesity-related diabetes, MOD) and a remaining group with no extreme characteristics (mild age-related diabetes, MARD). In our previous work we have further refined this MARD cluster into two clusters based on low (mild diabetes, MD) and high HDL-cholesterol levels (mild diabetes with high HDL-cholesterol, MDH). These clusters are based on five clinical variables: HbA_1c_, BMI, age, HDL-cholesterol and C-peptide [2]. We have also shown in our previous work that these clusters not only show differences in their clinical characteristics, but are also different on the lipid, protein and metabolite level [3].

Long non-coding RNAs (lncRNAs) are regulatory RNAs with a length of more than 200 nucleotides. The majority of the genome is comprised of non-coding RNAs, including lncRNAs [4], which are not translated into functional protein. lncRNAs are regulators of gene expression [5], for example via mRNA decay, which directly affects the production of proteins. There are multiple examples in which lncRNAs are involved in regulation of pathways associated with the development of diabetic complications [6–8].

While clusters differ in terms of clinical outcomes and diabetes progression, their differences on the molecular level are largely unclear. In our previous work we have shown that the insulin-resistant cluster has higher levels of branched-chain amino acids, diacylglycerol and triacylglycerol, and aberrant protein levels in plasma were enriched for proteins in the intracellular phosphoinositide 3-kinase (PI3K)/Akt pathway [3]. In addition, the obesity-related cluster showed higher cytokine levels. We hypothesise that these metabolic differences are also reflected in whole blood gene expression among individuals with type 2 diabetes assigned to one of the five clusters. To address this, we investigated differences in lncRNA and mRNA levels in whole blood of individuals with type 2 diabetes assigned to one of the five previously identified clusters. We also investigated the genetic influences on these mRNAs and the extent to which these lncRNAs and mRNAs play a suggestive, causal role in diabetes-relevant traits, such as lipid metabolism and anthropometric measures, using two-sample Mendelian randomisation (MR).

## Methods

### Participants

The Hoorn Diabetes Care System (DCS) cohort is an open prospective cohort started in 1998 with individuals with type 2 diabetes from the northwest part of the Netherlands. People visit the DCS annually to monitor their type 2 diabetes. Repeated measurements are collected as part of routine care during this visit, including anthropometric and lab measurements. Individuals in the Hoorn DCS cohort were asked to participate in the Hoorn DCS biobank in which, after obtaining informed consent, we also collected and stored blood samples for future research. All laboratory measurements were carried out on samples taken in a fasted state. Details of the laboratory measurements have been described in van der Heijden et al [9]. The study has been approved by the Ethical Review Committee of the VU University Medical Center, Amsterdam. Blood for RNA was collected in 2013 and 2014 from 1033 individuals who had participated in the biobank previously, without any specific selection criteria. From this group, 400 individuals were selected for RNA sequencing based on the following criteria: age at onset between 40 and 75 years; European descent; diabetes duration less than 10 years; and estimated eGFR > 30 ml/min.

### RNA isolation and sequencing

Details of the RNA isolation procedure and RNA sequencing have been described elsewhere [10]. In short, RNA was isolated from whole blood using the Direct-zol RNA MiniPrep (Zymo Research, Irvine, CA, USA). RNA sequencing libraries were generated using the Illumina Truseq v2 library preparation kit (Illumina, San Diego, CA, USA). Libraries were paired-end sequenced using the Illumina Hiseq2000. Reads were aligned to the genome using STAR (v2.3.0) [11]. Expression, as read count per RNA, was calculated using HTSeq (v0.6.1p1) with default settings based on the Ensembl v71 annotation (corresponding to GENCODE v16) [12, 13]. Counts were normalised using trimmed mean of M-values (TMM). Sex was confirmed using expression of *XIST* (chromosome X) and *UTY* (chromosome Y). The final dataset comprised expression levels of 391 individuals. RNAs with very low counts across all samples were filtered out.

### Clusters

Type 2 diabetes clusters were previously assigned by Slieker et al [2]. In short, individuals with type 2 diabetes in the DCS cohort were clustered based on five clinical variables: age at first visit (years); BMI (kg/m^2^); HbA_1c_ (mmol/mol); C-peptide (nmol/l) and HDL-cholesterol (mmol/l). Clustering was stratified by sex and were defined based on *k*-means. The following five clusters were defined: an insulin-deficient cluster (SIDD), an insulin-resistant cluster (SIRD), an obesity-related cluster (MOD) and mild clusters with low HDL-cholesterol levels (MD) and high HDL-cholesterol levels (MDH). The final dataset comprised expression levels and assigned clusters for 244 individuals.

### Blood cell fractions

Levels of neutrophils, lymphocytes, monocytes, eosinophils and basophils were measured with a UniCel DxH 800 Coulter Cellular Analysis System (Beckman Coulter, Brea, CA, USA) and the FC 500 Series system (Beckman Coulter) in all individuals with RNA-seq data. The *stats* R package (v4.3.0) was used for a linear model, adjusted for age and BMI to test for differences in white blood cell fractions across clusters.

### Statistical analysis

Power calculations were performed with the *ssizeRNA* R package (v1.3.2). For this we included the total number of genes, the dispersion parameter and mean count in the control group (mu). For the lncRNAs we have sufficient power (80%) to detect fold change 1.55 at a sample size of 26. For the mRNAs we have sufficient power (80%) to detect fold change 1.3 at a sample size of 26. Differential expression analysis was performed using a quasi-likelihood (QL) *F* test with the R package *edgeR* (v3.40.2). The base model was adjusted for the first three principal components of the blood cell fractions and technical covariates. We tested one cluster vs the other four clusters. RNA was considered differentially expressed if an observed difference between two conditions was statistically significant based on a false discovery rate (FDR)-adjusted *p* value below 0.05. A correlation heatmap between lncRNAs and mRNAs was made using the Spearman's rank-order correlation based on reads per kilobase per million mapped reads (RPKM) values. Lasso regression was performed testing each cluster separately with the R package *glmnet* (v4.1-3). lncRNA and mRNA *cis*-expression quantitative trait loci (eQTLs) were obtained from a publicly available dataset described by Võsa et al [14]. eQTLs were considered *cis*-eQTLs if the gene was <1 Mb from the SNP. *Cis*-eQTLs were considered significant at a *p* value below 5×10^−8^.

Traits associated with eQTLs were found in the publicly available genome-wide association study (GWAS) datasets IEU OpenGWAS [15]. The traits were divided into eight categories: anthropometry, lipid metabolism, cardiometabolic, metabolite/protein, white blood cell fractions, red blood cells, bone and other.

### Two-sample MR

To estimate the causal effect between lncRNAs/mRNAs and traits a two-sample MR analysis was performed using the TwoSampleMR R package (v0.5.6). To obtain independent SNPs, clumping was performed, removing SNPs in linkage disequilibrium (LD) *r*^2^ <0.001. Traits were used at a genome-wide level (*p*<5×10^−8^). The instrumental strength of each SNP was assessed using the *F* statistics = (β/SE)^2^. The mean *F* statistic of the SNPs used as instruments was reported and an *F* statistic >10 indicates a strong instrument [16]. For single instruments, the Wald ratio test was used: Wald estimates were calculated by dividing the SNP-outcome by the SNP-exposure. In the case of multiple instruments the inverse variance weighting (IVW) method was used, which uses information on all instruments. A causal association was statistically significant based on an FDR-adjusted *p* value below 0.05. For IVW estimates heterogeneity was calculated: a high heterogeneity indicates a high variance across instruments suggestive of invalid instruments. For visualisation of the MR results, a scatter plot for the effect of the SNPs on the exposure against the effect of the SNPs on the outcome was produced. A forest plot was used to visualise the estimates of multiple instruments. A funnel plot was plotted to visually assess heterogeneity and a leave-one-out plot to visualise the MR estimates when leaving one instrument out. We performed colocalisation analysis to assess pleiotropy with the *coloc* R package (v5.1.0.1). For this, we included all *cis*-acting SNPs associated with the gene before clumping and *p* value filtering. Next we determined the PP3 and PP4 posterior probabilities and calculated the extent of pleiotropy based on a threshold of PP4/(PP3+PP4) > 0.8 [17, 18]. We also performed colocalisation analyses as a sensitivity analyses for MR. Given that many loci are not limited to one signal, we dissected the individual signals using the *coloc.signals* function from the coloc R package (v5.1.0.1). For the colocalisation analyses we used all SNPs associated with the gene *in cis* before clumping and *p* value filtering. The *coloc.signals* function was used with the following settings: method: *conditioning*, mode: *allbutone* which allows testing of each signal without the influence of other signals, *p*=5×10^−8^, *r*^2^=0.001, maximum number of hits =5, prior probability P12 = 1×10^–5^. Colocalisation was defined as the posterior probability of H4 (PP.H4) higher than 0.8. Finally, reverse causality was assessed. For the above-described analyses we used the TwoSampleMR R package (v0.5.6). All analyses were performed using R statistics (v4.1.1). Figures were produced using the R package *NMF* (v0.23.0) and *ggplot2* (v3.3.5).

### External validation (IMI DIRECT study)

The Innovative Medicines Initiative Diabetes ResearCh on patient straTification (IMI DIRECT) study was used to validate the differentially expressed RNAs. In IMI DIRECT, individuals with type 2 diabetes from six cohorts were followed longitudinally. At baseline, clinical measures and multi-omics were measured, including RNA-seq (*n*=795). Details of the study design and the core characteristics are provided elsewhere [19, 20]. Six individuals were excluded based on DIRECT’s exclusion criteria and seven individuals could not be clustered due to missing data resulting in a dataset of 782 individuals. Individuals with RNA-seq data were assigned to one of the five clusters based on the cluster centres from DCS. Differential expression analysis was performed in the same way as described above. *p* values were Bonferroni adjusted based on the number of significant genes from the discovery dataset. A Bonferroni-adjusted *p* value below 0.05 was considered significant. Similarity between the effect size of the current study and IMI DIRECT study were calculated with the Pearson correlation coefficient.

## Results

Characteristics of individuals included in this study are given in electronic supplementary material (ESM) Table 1. The median (IQR) age of included individuals was 64.6 (57.7–70.3) years and 44.3% of the population was female. The population was overweight (median BMI: 29.3 [26.3–33.1]), with well controlled HbA_1c_ levels (median: 46.0 [42.0–52.3] mmol/mol; 6.4% [6.0– 6.9%]). The median age of type 2 diabetes diagnosis was 60.8 (54.0–66.5) years with a time since diagnosis of 3.3 (2.1–4.7) years and 16.0% of the study group smoked. As expected, the large majority (93.9%) were treated with metformin, 31.1% with sulfonylureas and 11.5% with insulin. Furthermore, 75.6% were treated with cholesterol-lowering drugs.

**Fig. 1:**
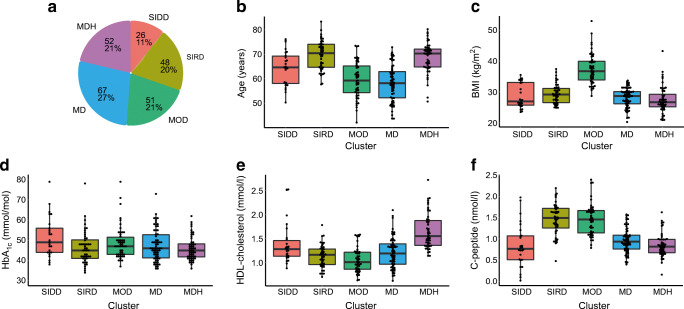
The characteristics of the five type 2 diabetes clusters. (**a**) Pie diagram of five clusters in the DCS Hoorn study (*n*=244). (**b**–**f**) The characteristics age, BMI, HbA_1c_, HDL-cholesterol and C-peptide were used to cluster type 2 diabetes patients in five clusters: SIDD, SIRD, MOD, MD and MDH. In total 244 individuals with type 2 diabetes participated in this study. Boxplot shows median, 25th percentile and 75th percentile

All individuals with type 2 diabetes participating in the Hoorn DCS biobank with sufficient data available for clustering (*n*=2953) were previously clustered based on the five clinical variables: age (years), BMI (kg/m^2^), HbA_1c_ (mmol/mol), HDL-cholesterol (mmol/l) and C-peptide (nmol/l). Characteristics of the subgroup used in this RNA-seq study (*n*=244) matched that of the larger group (Fig. 1a–f). We observed that the SIDD cluster defined by high HbA_1c_ represented 11% (*n*=26) of the individuals (ESM Table 1). Twenty per cent of the study population clustered to SIRD, 21% to MOD, 27% to MD and 21% to MDH.

### Blood cell fractions are not different across clusters

To examine whether different amounts of white blood cells in blood of individuals with type 2 diabetes are different across the clusters and therefore could possibly influence our subsequent analyses, we investigated blood cell fractions, including levels of neutrophils, lymphocytes, monocytes, eosinophils and basophils. In an unadjusted model we showed that neutrophil levels were lower in the MD cluster. The lymphocyte levels are higher in the SIRD cluster and lower in the MD cluster. The monocyte levels were differentially expressed in every cluster except in the MD cluster. In addition, the basophils are lower in the MDH cluster. We included the age of the individuals as an independent covariable owing to the fact that age can contribute to the content of white blood cells in the blood [21, 22]. In addition, BMI was included as an independent covariable owing to the fact that the white blood cell fractions are associated with BMI [23, 24]. In the adjusted model we show that the blood cell fractions are not differentially expressed across the clusters (ESM Table 2). Despite that the differences between the groups were relatively small, the first three principal components were taken along in the models (ESM Fig. 1).

### lncRNAs and mRNAs are differentially expressed in clusters

**Fig. 2:**
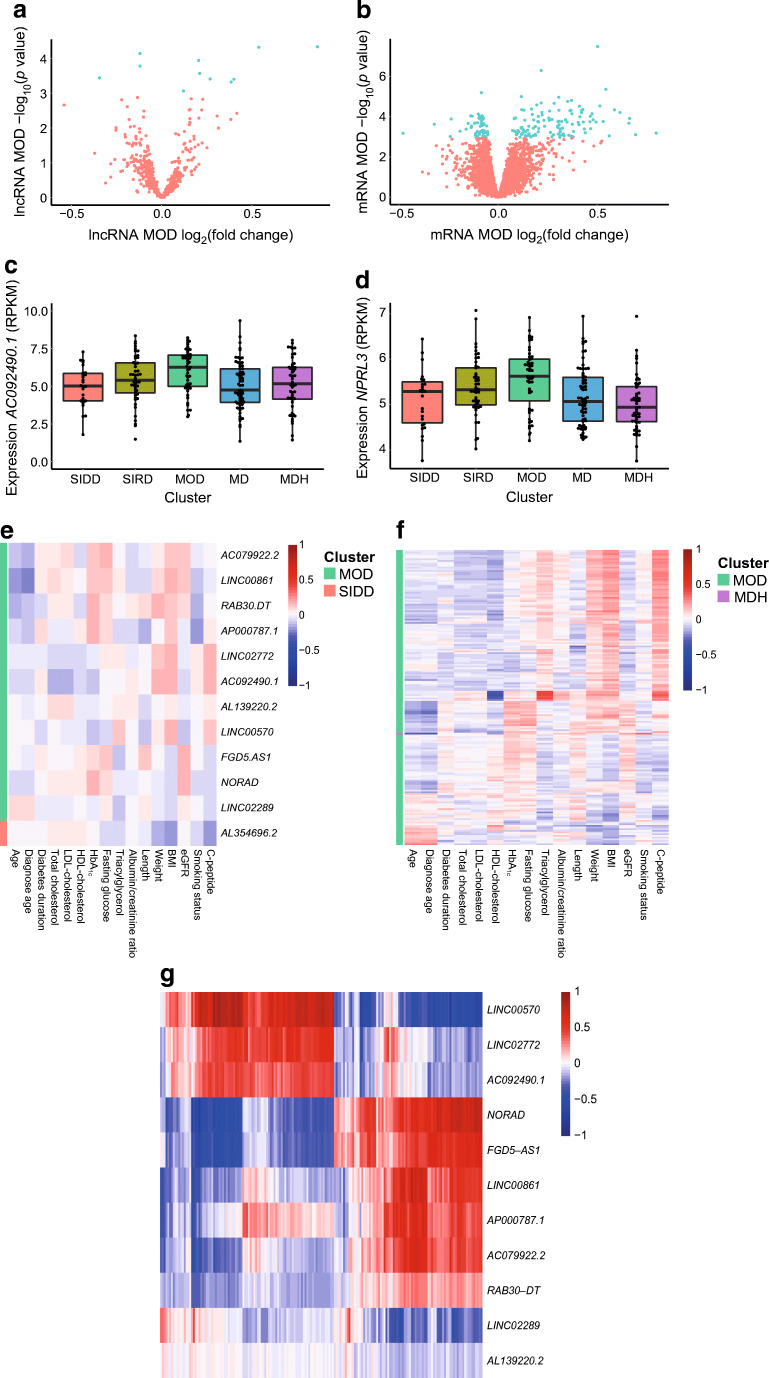
Differentially expressed RNAs in the MOD type 2 diabetes cluster. (**a**) Volcano plot of differentially expressed lncRNAs in the MOD cluster. (**b**) Volcano plot of differentially expressed mRNAs in the MOD cluster. (**c**) Expression (RPKM) of the lncRNA *AC092490.1* against the five clusters. Boxplot shows median, 25th percentile and 75th percentile. (**d**) Expression (RPKM) of *NPRL3* mRNA against the five clusters. Boxplot shows median, 25th percentile and 75th percentile. (**e**) Correlation heatmap of differentially expressed lncRNAs and clinical and biochemical characteristics. (**f**) Correlation heatmap of differentially expressed mRNAs and clinical and biochemical characteristics. (**g**) Correlation heatmap of differentially expressed lncRNAs and mRNAs in the MOD cluster. (**e**–**g**) The Spearman correlation coefficient is shown. Data were log_10_ transformed and *Z*-scaled

Out of the 574 lncRNAs expressed, we identified 12 lncRNAs associated with the clusters (ESM Fig. 2). Eight lncRNAs were upregulated in the MOD cluster compared with the other four clusters: *AC092490.1*, *LINC00570*, *RAB30-DT*, *AC079922.2*, *AP000787.1*, *LINC02772*, *AL139220.2* and *LINC00861* (Fig. 2a, ESM Fig. 3, ESM Table 3). The upregulated lncRNAs in the MOD cluster correlated with BMI and C-peptide, which is in line with the high BMI and low age and relatively lower levels of HbA_1c_ and HDL-cholesterol in the MOD cluster (Fig. 2e). Three lncRNAs were downregulated in the MOD cluster: *NORAD*, *FGD5-AS1* and *LINC02289* (Fig. 2a) and these lncRNAs correlated with age and HDL-cholesterol (ESM Fig. 4, Fig. 2e). On the basis of lasso regression, six out of 11 lncRNAs were selected for the MOD cluster (ESM Table 4). The lncRNA *AL354696.2* was upregulated in the SIDD cluster and was also selected for this cluster (ESM Table 4). The MOD cluster was also the cluster with the most significant differentially expressed mRNAs. In total 175 mRNAs were differentially expressed in the MOD cluster, 118 were upregulated and 57 downregulated (Fig. 2b, ESM Fig. 5, ESM Table 5). The upregulated mRNAs in the MOD cluster correlated with BMI, weight and C-peptide, which is in line with the high BMI and low age and relatively lower levels of HbA_1c_ and HDL-cholesterol in the MOD cluster. The downregulated mRNAs in the MOD cluster correlated with age and HDL-cholesterol (Fig. 2f). On the basis of lasso regression we found that 18 out of 175 mRNAs were selected for the MOD cluster (ESM Table 6). *GPR15* was the only mRNA different in the MDH cluster and was downregulated and correlated with length (Spearman’s ρ 0.19) and HbA_1c_ (Spearman’s ρ 0.11). *GPR15* is a chemoattractant receptor that regulates T cell migration and immunity [25]. *GPR15* has previously been described to be induced in individuals that smoke compared with non-smokers [26, 27]. Indeed, in the MDH cluster the percentage of smokers (percentage 3.8%) was much lower compared with other clusters (percentage 16%). Interestingly, this is particularly low compared with the MD cluster where 28.4% smoked. To verify that *GPR15* expression is influenced by smoking status, *GPR15* was plotted against individuals that never smoked, former smokers and smokers (ESM Fig. 6). We observed that the expression of *GPR15* was very low for individuals who had never smoked compared with individuals who were former smokers (*p*=2×10^−9^) and even more to smokers (*p*=1×10^−22^). After adjustment for BMI, the differentially expressed mRNAs are completely eliminated in the MOD cluster. Adjustment for age and sex resulted in similar differentially expressed lncRNAs and mRNAs (ESM Fig. 7).

Next, we investigated the pairwise correlation between the differently expressed lncRNAs and mRNAs in the MOD cluster (ESM Table 7). We observed that lncRNAs (*LINC00570*, *LINC02772*, *AC092490.1*, *NORAD*, *FGD5-AS1*, *LINC00861*, *AP000787.1* and *AC079922.2*) strongly correlated with multiple mRNAs (Fig. 2g). The top ten positively correlated lncRNAs and mRNAs had a correlation coefficient between 0.93 and 0.84 and the top negatively correlated ranged from −0.76 and −0.61 (ESM Table 8). This correlation is visualised in the scatterplot of the top three positive and negative correlations (ESM Fig. 8). Pathway analysis did not yield any significantly enriched pathways (data not shown). Interestingly, genes identified in the current study showed an overlap (110 genes) with BMI-associated genes [28]. Of note, in the current study we identify 65 additional genes that were not observed in this external study (ESM Fig. 9, ESM Table 9).

### External validation (IMI DIRECT study)

**Fig. 3:**
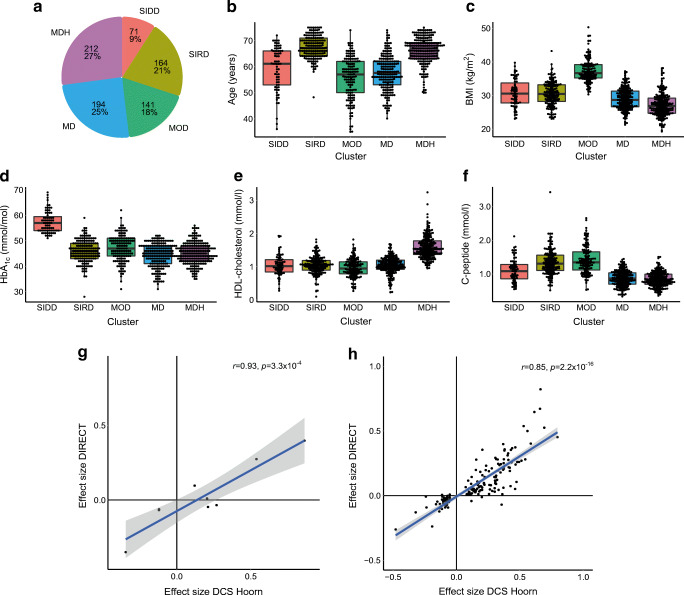
Validation in the IMI DIRECT study. (**a**) Pie diagram of five clusters in the IMI DIRECT study (*n*=782). (**b–f**) Characteristics of the five type 2 diabetes clusters in the IMI DIRECT study: age, BMI, HbA_1c_, HDL-cholesterol and C-peptide. Boxplot shows median, 25th percentile and 75th percentile. (**g**) Effect size plot of differentially expressed lncRNAs from the current study (DCS Hoorn) and the IMI DIRECT study. (**h**) Effect size plot of differentially expressed mRNAs from the current study (DCS Hoorn) and the IMI DIRECT study

Characteristics of individuals from the IMI DIRECT study are given in ESM Table 1. Characteristics of the IMI DIRECT study matched that of the discovery set (Fig. 3a–f). In the current study (DCS Hoorn), 11 lncRNAs were differentially expressed in the MOD cluster. Two of these were not available in the RNA-seq data of the IMI DIRECT study, six were validated based on the Bonferroni-adjusted *p* value and three were not validated (ESM Table 10). The lncRNA found to be upregulated in the SIDD cluster in the current study was not available in the IMI DIRECT data. The nine lncRNAs available in the IMI DIRECT study had similar effect sizes in the current study (DCS Hoorn) and the IMI DIRECT study (Pearson’s ρ 0.93, *p*=3.3×10^-4^) (Fig. 3g).

In total 175 mRNAs were found to be differentially expressed in the MOD cluster of the current DCS Hoorn study, one of these was not available in the RNA sequencing data of the IMI DIRECT study, 119 mRNAs were validated based on the Bonferroni-adjusted *p* value whereas 55 were not validated. *GPR15* was also downregulated in the MDH cluster, based on the Bonferroni-adjusted *p* value, in the IMI DIRECT study (ESM Table 11). One hundred and seventy-five mRNAs had a similar effect size in the current study (DCS Hoorn) and the IMI DIRECT study (Pearson’s ρ 0.85, *p*=2.2×10^-16^) (Fig. 3h). We continued the analysis with the Bonferroni significant lncRNAs and mRNAs in both the discovery and replication cohorts.

### Differentially expressed mRNAs in the MOD cluster may have a causal role

**Fig. 4:**
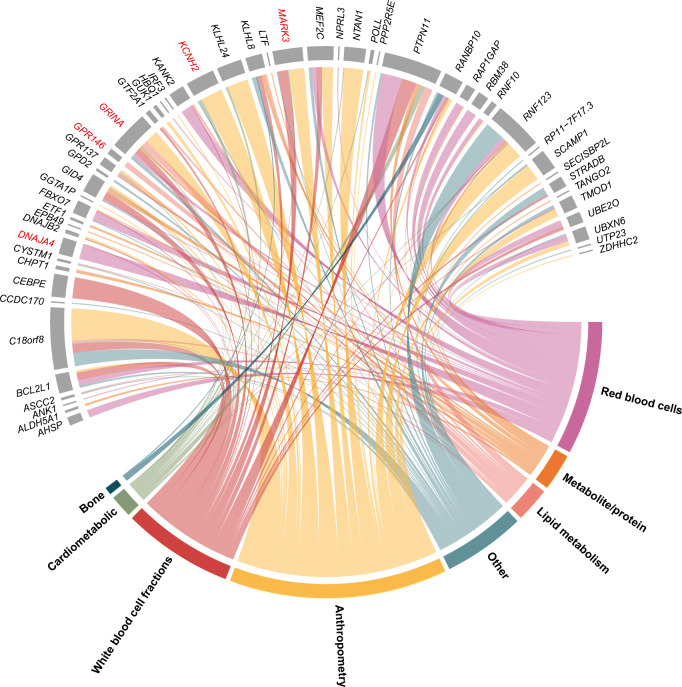
Chord diagram of causal associations with strong MR evidence. Two-sample MR test performed on lncRNAs and mRNAs in the MOD cluster and related traits found with the IEU OpenGWAS database. The categories to which the traits belong are visible on the bottom. The following mRNAs are highlighted in red: *GRINA*, *MARK3*, *KCNH2*, *GPR146* and *DNAJA4*. These mRNAs are highlighted because they are based on multiple instruments (≥2) instead of the Wald ratio (1 SNP) and the most significant (lowest *p* value) for each category of traits = red blood cells, lipid metabolism, anthropometry and white blood cell fractions

To investigate the role these lncRNAs and mRNAs may have on related traits, *cis*-expression quantitative trait loci (*cis*-eQTLs) were selected from a previously published dataset [14]. The *cis*-eQTLs were compared with published GWASs in the IEU OpenGWAS database [15]. In total, one *cis*-eQTL was associated with the expression of one lncRNA and 207 *cis*-eQTLs were associated with the expression of 103 mRNAs after clumping SNPs that are in LD (*r*^2^ <0.001). The range of the mean *F* statistics of the SNPs used as instruments was 43.2–2113.4, indicating strong instruments. Multiple mRNAs were found to be associated with anthropometric, lipid metabolism and blood cell fraction traits. Next, we wanted to estimate the causal association these mRNAs may have on the traits found with the IEU OpenGWAS database. A two-sample MR test was performed (ESM Table 12). A chord diagram based on FDR-adjusted *p* values of the MR results on the lncRNAs and mRNAs and related traits is shown in Fig. 4. In total 52 mRNAs were shown to have a suggestive causal effect on 217 traits that related to anthropometric, lipid and blood cell fraction traits.

Twenty-three mRNAs were causally associated with anthropometric traits (Fig. 4). We found that *KCNH2* showed the strongest causal effect on anthropometric traits: the trunk fat-free mass and trunk predicted mass (IVW, 2 SNPs, *p*=8×10^−22^) (ESM Table 12). Higher genetically determined levels of *KCNH2* are suggested to reduce trunk fat-free mass (ESM Fig. 10a, ESM Fig. 11). We found no evidence for heterogeneity (*Q*=0.74, *p*=0.39), pleiotropy (5×10^−5^) and reverse causality (*p*=0.96) (ESM Table 12). This causal effect was not supported by colocalization analyses (PP.H4=0) (ESM Table 12). Six mRNAs showed a causal effect on BMI, however five causal associations are based on one instrument.

Ten mRNAs were causally associated with all lipid metabolism traits, some mRNAs were causally associated with almost all lipid metabolism traits, and others were only causally associated with one type of lipid trait, for example LDL-cholesterol (Fig. 4). We observed that *GRINA* had the strongest causal effect on LDL-cholesterol (IVW, 2 SNPs, *p*=2×10^−31^) (ESM Fig. 12). Higher genetically determined levels of *GRINA* are suggested to reduce LDL-cholesterol levels in plasma (ESM Fig. 10b, ESM Fig. 12). This causal effect was supported by colocalization analysis (PP.H4=0.99) (ESM Table 12). We found no evidence for heterogeneity (*Q*=0.30, *p*=0.58), pleiotropy (4×10^−3^), nor for reverse causality (*p*=0.92) (ESM Table 12). *GRINA* was found to have a strong correlation with two lncRNAs, one of which was also upregulated in the MOD cluster: *LINC00570* (Spearman’s ρ 0.54) and one downregulated in the MOD cluster: *NORAD* (Spearman’s ρ −0.57) (ESM Fig. 13). GRINA also has a suggested causal effect on platelets (IVW, 2 SNPs, *p*=2×10^−82^) (ESM Table 12). Furthermore, *GPR146* had the strongest causal effect on HDL-cholesterol (IVW, 2 SNPs, *p*=2×10^−15^) (ESM Fig. 14). Higher genetically determined levels of *GPR146* are suggested to induce higher levels of HDL-cholesterol in plasma (ESM Fig. 10c, ESM Fig 14). This causal effect was supported by colocalization analysis (PP.H4=0.99) (ESM Table 12). Again we found no evidence for heterogeneity (*Q*=0.86, *p*=0.35), pleiotropy (2×10^−4^) and reverse causality (*p*=0.81) (ESM Table 12).

Nineteen mRNAs had a causal effect on white blood cell fractions (Fig. 4). *CEBPE* has a causal effect on almost every white blood cell (basophil, eosinophil, granulocyte, monocyte and neutrophil), based on the Wald ratio with one instrument (ESM Table 12). These causal effects were supported by colocalization analyses (PP.H4>0.8) (ESM Table 12). We could not test for heterogeneity based on one instrument; however, we found evidence for reverse causality on eosinophil (*p*=5×10^−26^) and neutrophil fractions (*p*=8×10^−11^) (ESM Table 12). Furthermore, strong evidence for pleiotropy was found. *MARK3* showed a causal effect on monocyte cell count (IWV, 3 SNPs, *p*=2×10^−159^) (ESM Fig. 15). This was supported by colocalization analysis (PP.H4=0.99) (ESM Table 12). Higher genetically determined levels of *MARK3* are suggested to reduce monocyte levels in plasma (ESM Fig. 10d, ESM Fig. 15). We found no evidence for heterogeneity (*Q*=0.43, *p*=0.80) nor reverse causality (*p*=0.99). However, we found evidence for pleiotropy (0.99) (ESM Table 12). We also found 20 mRNAs that had a causal association with red blood cells (Fig. 4). Based on the Wald ratio with one instrument *KANK2* had the highest causal effect on reticulocyte traits (*p*=4×10^−227^); however, we found evidence for pleiotropy (0.95) and reverse causality (*p*=0.01) (ESM Table 12). With the IVW method based on three instruments we found that *DNAJA4* has a causal effect on the reticulocyte count (*p*=4×10^−18^) (ESM Fig. 16). Higher genetically determined levels of *DNAJA4* are suggested to induce higher levels of reticulocytes (ESM Fig. 10e, ESM Fig. 16). Here we found no evidence for heterogeneity (*Q*=1.53, *p*=0.47), pleiotropy (9×10^−7^) nor reverse causality (*p*=0.95). However, this suggested causal effect was not supported by colocalisation analysis (PP.H4=0) (ESM Table 12).

## Discussion

The aim of this study was to identify differentially expressed RNAs in blood of patients with type 2 diabetes in five previously defined clusters. In the current study, we show that lncRNAs and mRNAs are differentially expressed primarily in MOD, and much less often in other clusters. In total, 11 lncRNAs and 175 mRNAs were differentially expressed in the MOD cluster. In addition, the lncRNA *AL354696.2* was upregulated in the SIDD cluster and *GPR15* mRNA was downregulated in the MDH cluster. Of those, six lncRNAs and 120 mRNAs were validated in the IMI DIRECT study. A strong correlation was observed between the lncRNAs and mRNAs found in the MOD cluster, suggesting a possible relation between them. Interestingly, we showed that the expression of specific genes may have a causal role on multiple traits linked to anthropometrics, lipid metabolism and blood cell fractions.

Using the five clusters, almost all identified RNAs showed aberrant expression in the MOD cluster. We show that this subgroup, comprised of people with diabetes and a high BMI, have an altered blood transcriptome profile compared with the other clusters. A possible explanation for the profound differences in MOD vs other clusters may be that obesity is associated with low-grade inflammation [29, 30]. Even though this generally occurs within metabolic tissues, our results suggest that changes may also occur in the expression levels of circulating blood cells, which we observed previously as well [10].

The identified lncRNAs correlated with the expression of specific mRNAs. It has previously been described that lncRNAs regulate a wide range of biological processes through their crosstalk with miRNAs that, in turn, regulate mRNAs [31]. This suggests that target mRNA would play a role in the same pathway, but we did not observe such an enrichment. However, in the two-sample MR we showed that many of the identified genes do not play a role in a single pathway but in several very distinct processes, for example lipid metabolism and blood cell fractions.

A two-sample MR analysis was used to evaluate a possible causal relation between whole blood RNAs that were differentially expressed in the MOD cluster and traits. For the anthropometric traits, we found a suggestive causal relationship for 23 mRNAs. Among them, *KCNH2* was found to be upregulated in the MOD cluster and is suggested to reduce the trunk fat-free mass. The fat-free mass is a marker of body muscle development. *KCNH2* encodes a voltage-activated potassium channel that has been mainly indicated as playing a role in long QT syndrome [32, 33]. Obesity is associated with long QT syndrome, where it is suggested to decrease expression of potassium channels [34].

Twelve mRNAs were suggested to have a causal effect on lipid metabolism. We found that higher *GRINA* expression is suggested to reduce LDL-cholesterol levels in plasma. *GRINA* is also suggested to have a causal effect on platelets (ESM Table 12). This indicates pleiotropy; however, LDL-cholesterol and platelets interact with each other. Oxidised (ox)-LDL leads to platelet activation and the activated platelets produce reactive oxygen species, which can oxidise LDL-cholesterol again [35]. Jiménez-González et al suggested that *GRINA* may regulate genes involved in lipid and cholesterol synthesis [36]. For example, *GRINA* has been shown to interact with *SREBP1* in *Caenorhabditis elegans* [37, 38]. *SREBP1* and *SREBP2* are both regulators of lipid biosynthesis [39]. They control the expression of several enzymes necessary for cholesterol, fatty acid, triacylglycerol and phospholipid synthesis [40]. Interestingly, *SREBP1* can also activate gene expression of *FDPS* and *FDFT1*, which were also found to be upregulated in the MOD cluster (ESM Table 5) [41–47]. *FDPS* encodes a gene that facilities the formation of farnesyl pyrophosphate, which is a key intermediate in cholesterol biosynthesis [48]. The *FDFT1* gene also plays a role in a later stage of the sterol and cholesterol biosynthesis [49]. It encodes a membrane-associated enzyme that is the first specific enzyme in cholesterol biosynthesis, catalysing the dimerisation of two molecules of farnesyl diphosphate in a two-step reaction to form squalene [50]. This suggests that multiple aberrant RNAs in the MOD cluster play a role in cholesterol synthesis.

One of the striking genes was *GPR146*, for which we show that higher expression was suggested to increase HDL-cholesterol levels in plasma. While higher HDL-cholesterol levels in the MOD cluster may seem counterintuitive—HDL-cholesterol levels are lowest in the MOD cluster—previous reports have shown that there is an overall change in lipid homeostasis in relation to *GPR146*. Specifically, *GPR146* regulates plasma cholesterol levels through the sterol regulatory element-binding protein 2 (SREBP2) signalling pathway and ERK signalling [51]. Increased expression of *GPR146* has been shown to be associated with increased plasma total cholesterol levels in humans [52–54]. In line with these findings Yu et al found that the HDL-cholesterol, LDL-cholesterol, VLDL-cholesterol and total cholesterol levels were reduced in *GPR146* deficient mice. The latter study suggests that *GPR146* may be a new therapeutic target to treat hypercholesterolemia and atherosclerotic cardiovascular disease, whereby the cholesterol levels are too high and build up in the artery wall [51].

We identified a causal relationship between mRNAs and white blood cell fractions. *CEBPE* was found to be causally associated with almost all white blood cell fractions. *CEBPE* is essential for functional maturation of granulocyte–monocyte progenitor cells [55]. However, strong pleiotropy was observed, which indicates that a single genetic variant influences multiple traits.

In addition, 20 mRNAs were causally associated with red blood cell traits. However, in these groups we also observed the largest reverse causality. This suggests that differences in blood cell fractions affects gene expression in the MOD cluster. Nonetheless, we observed minimal differences in white blood cell fractions between the clusters and adjusted for them, although the differences may be more prominent in specific blood cell subtypes.

We previously looked at plasma metabolomic, lipidomic and proteomic data in the five clusters [3]. We showed that the insulin-resistant cluster (SIRD) showed the most aberrant molecular signature with the highest lipid levels. However, we did not observe such an enrichment in the SIRD cluster in the current study. We also showed previously that the obesity-related cluster has a similar molecular signature to the SIRD cluster, but with higher cytokine levels. Interestingly, growth hormone receptor, which we previously showed to be upregulated in the MOD cluster, has been shown to interact with *PTPN11* [56, 57], which was differentially expressed in the MOD cluster in the current study. In addition, the lipid profile and differentially expressed proteins strongly reduced after adjustment for BMI, which is in line with our findings. This study shows that clustering individuals with type 2 diabetes can identify underlying novel biological insights into the diverse pathophysiological mechanisms and underlying phenotypes of the clusters, which we show also occurs in circulating blood cells. Here we show that obesity or higher BMI is the driving force behind the differentially expressed RNAs in the circulating blood cells. However, based on the findings by Huan et al, where they found mRNA expression levels associated with BMI, 110 mRNAs overlap with the 175 mRNAs found to be differentially expressed in the MOD cluster in the current study [28]. Moreover, 65 mRNAs did not overlap between the BMI-associated mRNAs from Huan et al and the 175 mRNAs found to be differentially expressed in this study, and therefore they seem to be type 2 diabetes MOD cluster-specific.

*GPR15* was downregulated in the MDH cluster. It has been described that tobacco smoking is a strong inducer of *GPR15* expression in peripheral blood [58]. In the MDH cluster on average fewer individuals smoke than the other clusters, which was associated with downregulated expression of *GPR15*. Therefore, it seems that low *GPR15* expression seen in the MDH cluster results from the lower smoking levels in this group. Individuals in the MOD cluster have on average the highest BMI, which could be the reason for the higher numbers of differently expressed RNAs in that cluster. We found different mRNAs to have a causal effect on multiple lipid metabolism traits such as total cholesterol, LDL-cholesterol and HDL-cholesterol; although this might seem to indicate that differentially expressed mRNAs in the MOD cluster are not only driven by the high BMI, this difference was completely eliminated after adjustment for BMI.

We show that the diabetes subgroup comprised people with a high BMI (the MOD cluster) have an altered blood transcriptome profile compared with the other clusters, which supports the idea of a different underlying pathophysiological process for each cluster. It has been suggested that clustering individuals with type 2 diabetes based on the five variables may not give a greater clinical utility than modelling clinical features directly [59]. Indeed, in part, the observed changes will be driven by the high BMI in the cluster. Nonetheless, we and others have shown that the other clusters also have their own genetic, metabolomic, proteomic and epigenetic signatures [3, 60, 61]. Despite its caveats, the clusters may help to further stratify people with diabetes and provide a more holistic view of type 2 diabetes [62].

This study has several strengths and weaknesses. Strengths include the use of a well phenotyped cohort. The external validation of the results found in this study, which further establishes the heterogeneity of these type 2 diabetes clusters, is also a strength. A weakness is that the sample size for each of the clusters was relatively small to detect small differences between clusters. In the SIDD cluster, we only had enough power to detect larger effect sizes. However, we did not see a relationship between the number of individuals in a cluster and the number of differentially expressed RNAs. Another potential weakness was the use of a relative complex tissue, where results may be driven by differences in blood cell fractions. However, we mitigated this by adjusting for blood cell composition based on measured blood cell fractions. We performed MR, which is independent of the confounders that influence whole blood gene expression. Also, we observed that a large number of the causal associations are based on one instrument, which is less reliable then using multiple instruments. However, we mainly looked at causal associations based on two or more instruments, which increases reliability, explained variance and power. In addition, we used colocalization analysis as a sensitivity analyses for the significant MR results.

### Conclusion

In the current study we identified 11 lncRNAs and 175 mRNAs to be differentially expressed in the MOD cluster. Strong correlation was observed between lncRNAs and mRNAs differentially expressed in the MOD cluster. Differentially expressed genes were validated for the large part in the IMI DIRECT study. Multiple mRNAs are suggested to have a causal effect on multiple traits linked to anthropometrics, lipid metabolism and blood cell fractions. Together, our results show that individuals in the MOD cluster show aberrant RNA expression of genes that have a suggested causal role on multiple diabetes-relevant traits.

## Supplementary Information

ESM (PDF 1083 kb)

ESM Tables (XLSX 3589 kb)

## Data Availability

Individual level data will be available upon request by contacting the corresponding author but access to data must be granted by the respective steering committee.

## Abbreviations

DCS: Diabetes Care System
eQTL: Expression quantitative trait locus
FDR: False discovery rate
GWAS: Genome-wide association study
IMI DIRECT: Innovative Medicines Initiative DIabetes REsearCh on patient straTification
IVW: Inverse variance weighting
LD: Linkage disequilibrium
lncRNA: Long non-coding RNA
MARD: Mild age-related diabetes
MD: Mild diabetes
MDH: Mild diabetes with high HDL-cholesterol
MR: Mendelian randomisation
MOD: Mild obesity-related diabetes
PP3/4: Posterior probability 3/4
SAID: Severe autoimmune diabetes
SIDD: Severe insulin-deficient diabetes
SIRD: Severe insulin-resistant diabetes
QL: Quasi-likelihood
