# Supplementary material for: Altered blood gene expression in the obesity-related type 2 diabetes cluster may be causally involved in lipid metabolism: a Mendelian randomisation study
Source: Diabetologia. 2023 Feb 24;66(6):1057–70. doi: 10.1007/s00125-023-05886-8 (PMC10163084; doi:10.1007/s00125-023-05886-8)
Supplement: Supplementary file 1 — (PDF 1083 kb) [file 125_2023_5886_MOESM1_ESM.pdf]

SUPPLEMENTARY DATA

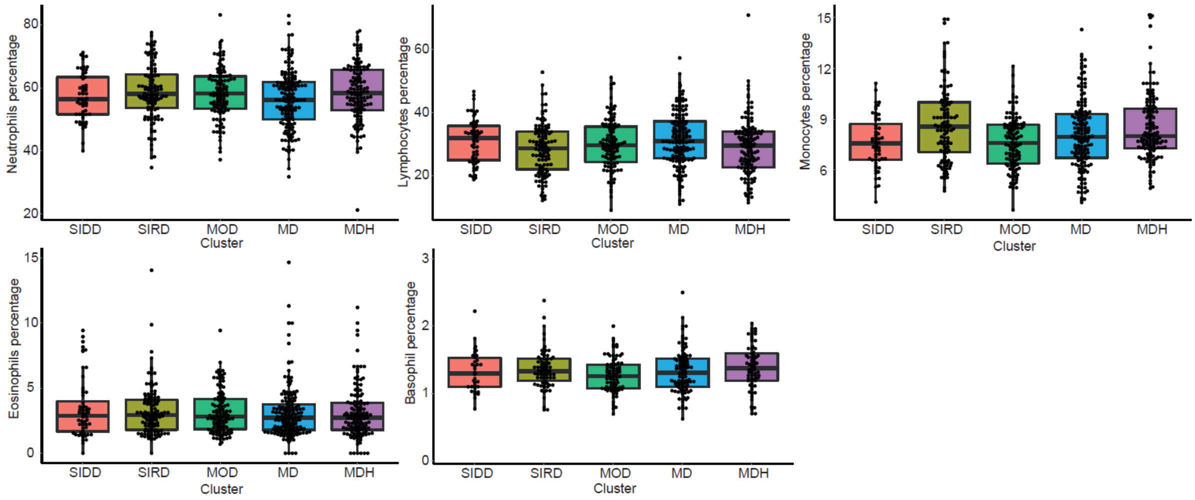

ESM Fig. 1 Percentage of white blood cells in blood of individuals in the five clusters of T2D.
Percentage of neutrophils, lymphocytes, monocytes, eosinophils and basophils in blood of individuals with T2D in the five clusters: SIDD, SIRD, MOD, MD and MDH

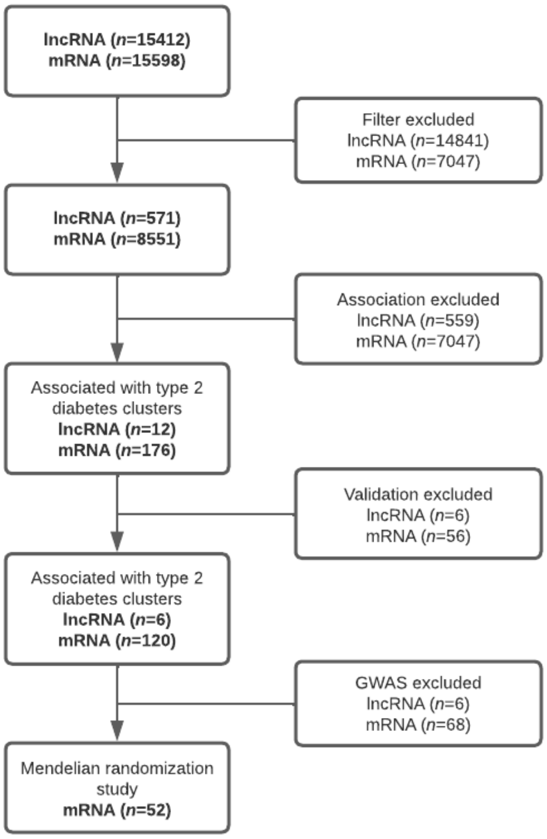

ESM Fig. 2 Overview of the study

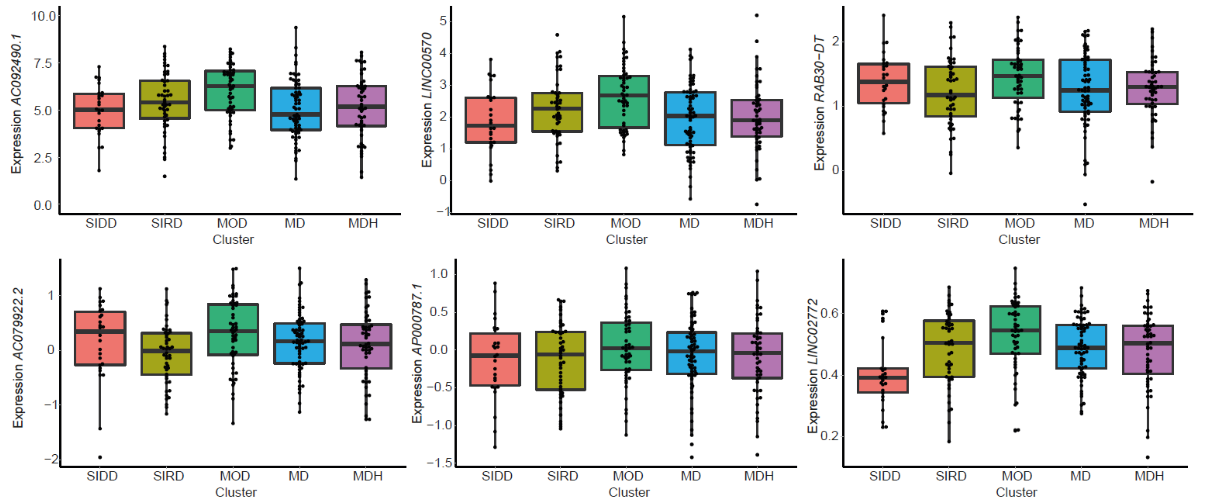

ESM Fig. 3 Upregulated long non-coding RNAs in the MOD cluster. Top six upregulated long non-coding RNAs in the MOD cluster compared to the other clusters. Long non-coding RNA *AC092490.1*, *LINC00570*, *RAB30-DT*, *AC079922.2*, *AP000787.1*, *LINC02772*.

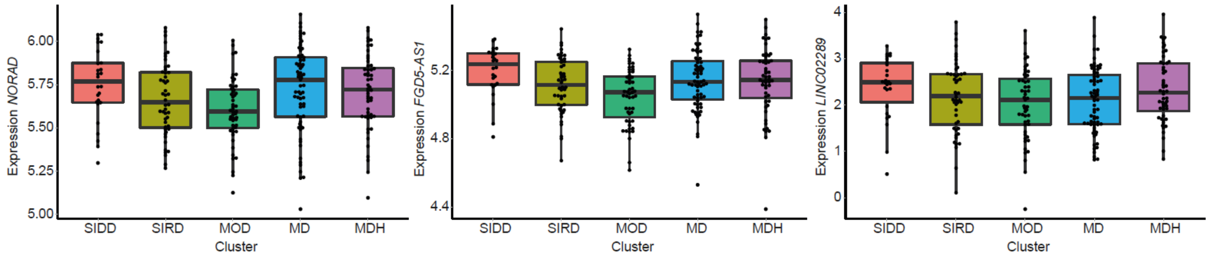

ESM Fig. 4 Downregulated long non-coding RNAs in the MOD cluster. Downregulated long non-coding RNAs in the MOD cluster compared to the other clusters. Long non-coding RNA *NORAD*, *FGD5-AS1* and *LINC02289* can be seen.

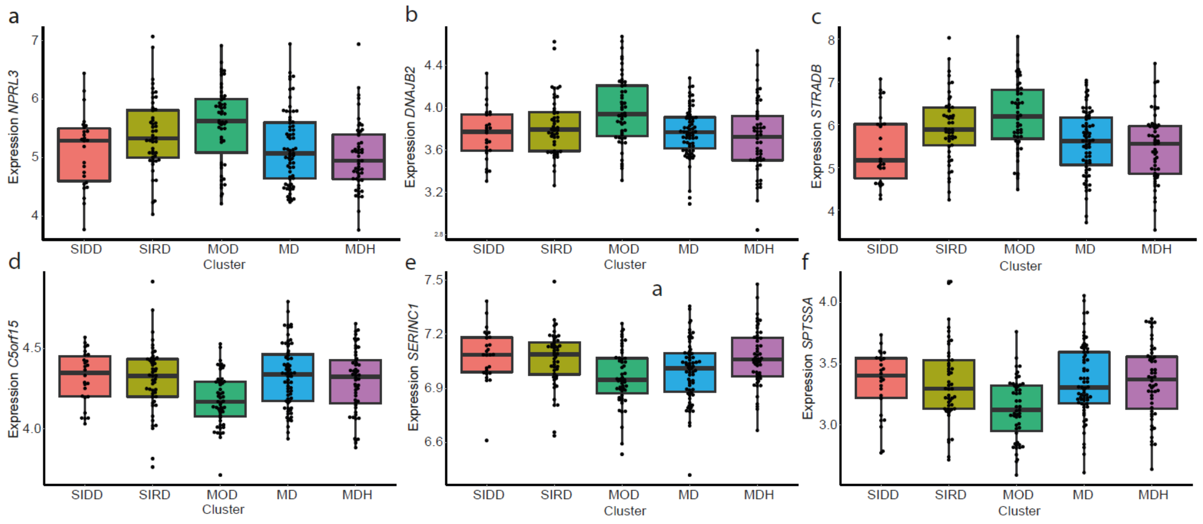

ESM Fig. 5 Differentially expressed mRNAs in the MOD cluster. **A**, **B** and **C** top three upregulated mRNAs in the MOD cluster compared to the other clusters: *NPRL3*, *DNAJB2* and *STRADB*. **D**, **E** and **F** top three downregulated mRNAs in the MOD cluster compared to the other clusters: *C5orf15*, *SERINC1* and *SPTSSA*.

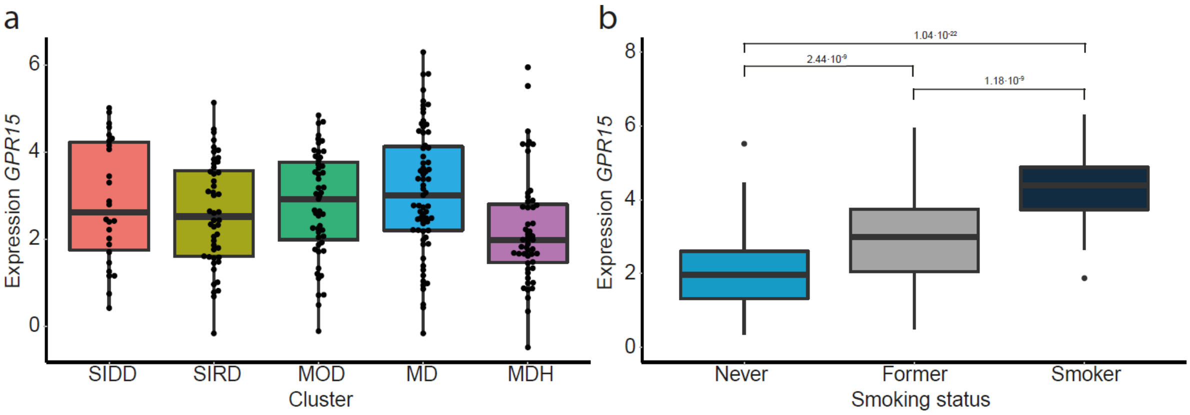

ESM Fig. 6 Plot of the expression of *GPR15* against cluster and smoking status.
A. Expression of *GPR15* against cluster: SIDD, SIRD, MOD, MD and MDH. B. Expression of *GPR15* against smoking status: never, former and smoker. Adjusted P-values are visible.

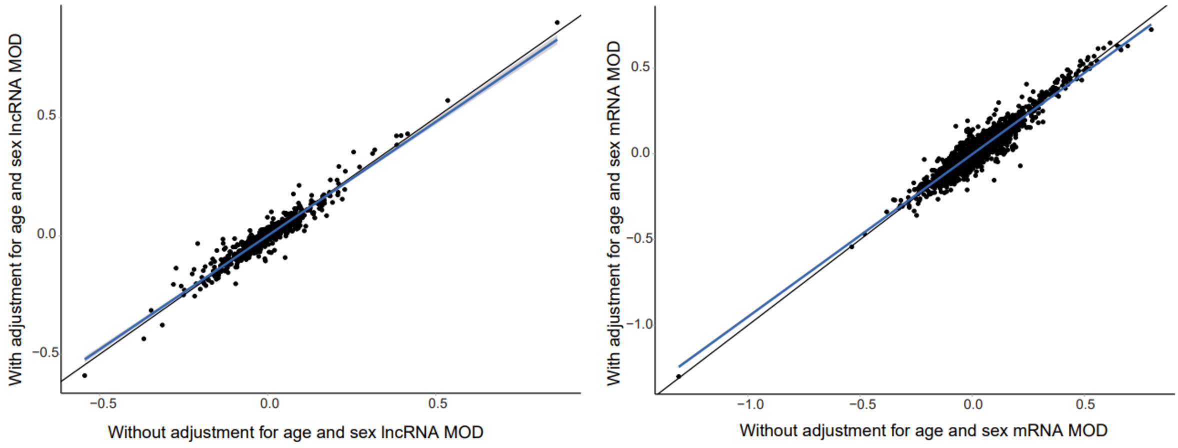

ESM Fig. 7 Adjustment for age and sex
Adjustment for age and sex resulted in similar differentially expressed lncRNAs and mRNAs in the MOD cluster

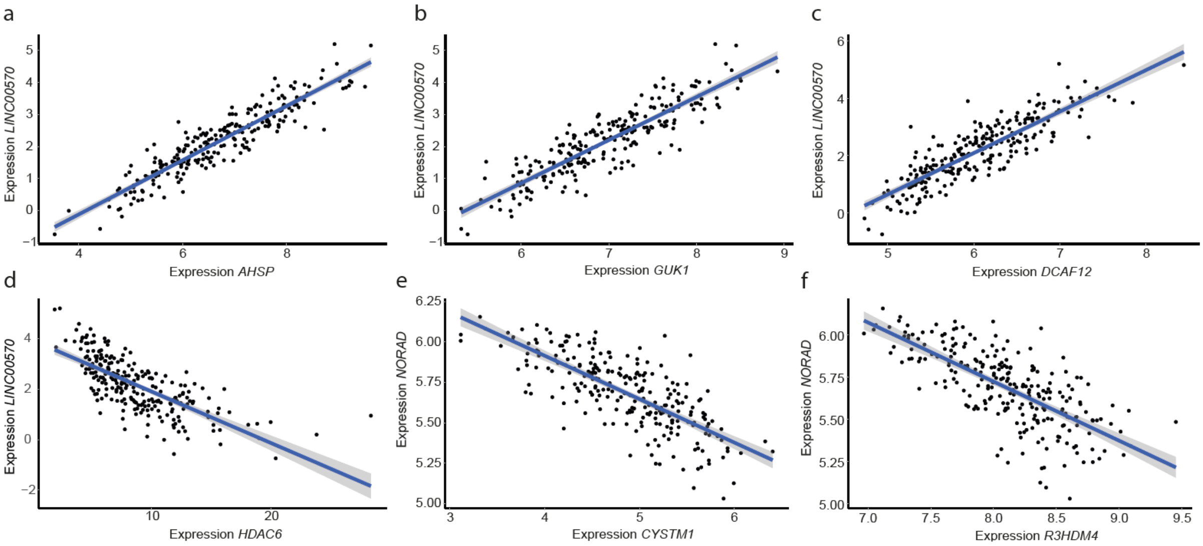

ESM Fig. 8 Scatterplot expression of the top three positive and three negative correlated lncRNAs and mRNAs. **A.** correlation *LINC00570* and *AHSP*. Spearman correlation coefficients 0.925. **B.** Correlation *LINC00570* and *GUK1*. Spearman correlation coefficients 0.880. **C.** correlation *LINC00570* and *DCAF12*. Spearman correlation coefficients 0.854. **D.** correlation *LINC00570* and *HDAC6*. Spearman correlation coefficients -0.761. **E.** correlation *NORAD* and *CYSTM1*. Spearman correlation coefficients -0.755. **F.** correlation *NORAD* and *R3HDM4*. Spearman correlation coefficients -0.738.

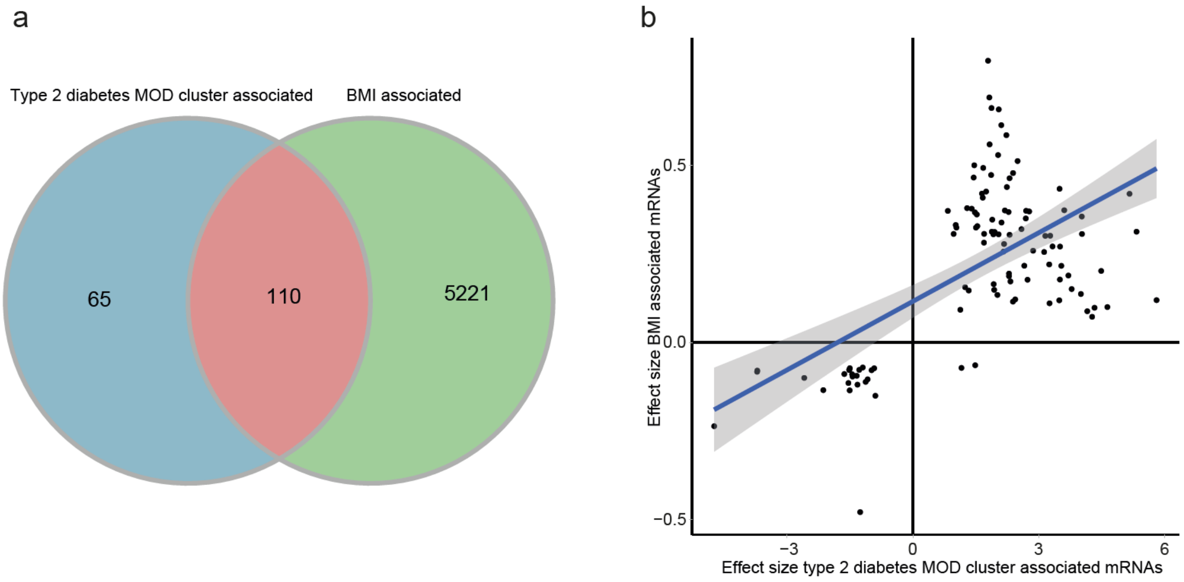

ESM Fig. 9 Overlap mRNAs type 2 diabetes MOD cluster and BMI associated. **A.** Venn diagram of overlapping mRNAs between type 2 diabetes MOD cluster and BMI associated. **B.** Effect size plot of 110 overlapping mRNAs.

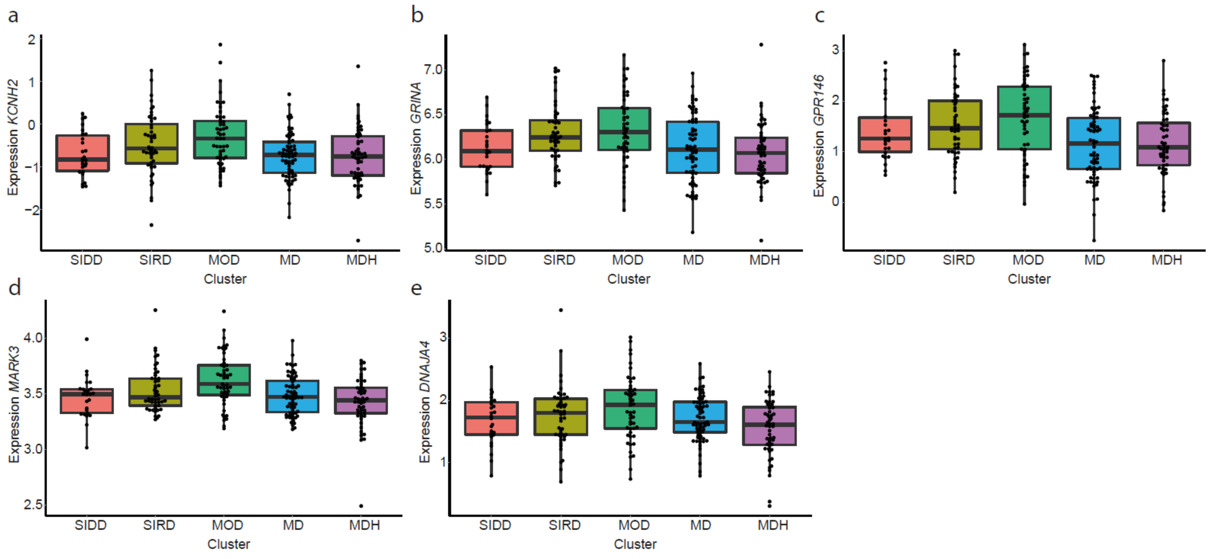

ESM Fig. 10 Upregulated mRNAs in the MOD cluster. **A.** Upregulated mRNA *KCNH2* in the MOD cluster. **B.** Upregulated mRNA *GRINA* in the MOD cluster. **C.** Upregulated mRNA *GPR146* in the MOD cluster. **D.** Upregulated mRNA *MARK3* in the MOD cluster. **E.** Upregulated mRNA *DNAJA4* in the MOD cluster.

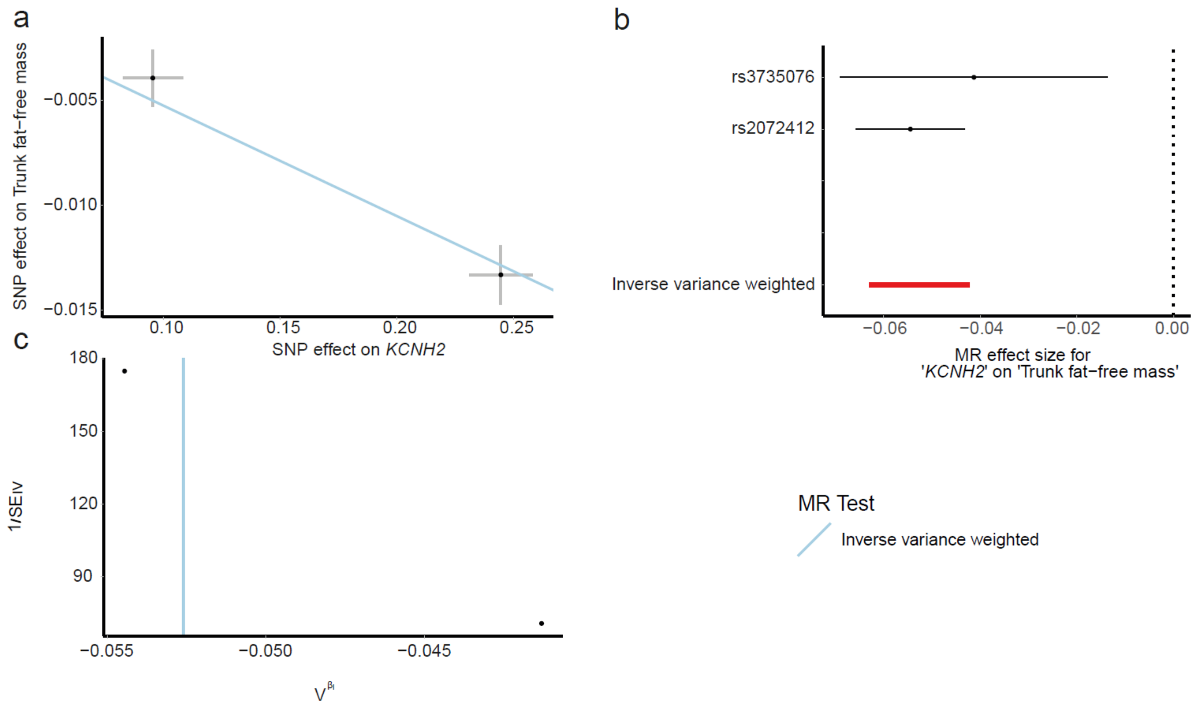

ESM Fig. 11 Plots MR base results *KCNH2* on trunk fat-free mass. **A.** Scatterplot of SNP effect *KCNH2*. **B.** Forest plot of MR effect size *KCNH2* on trunk fat-free mass. **C.** Funnel plot for distribution of instruments.

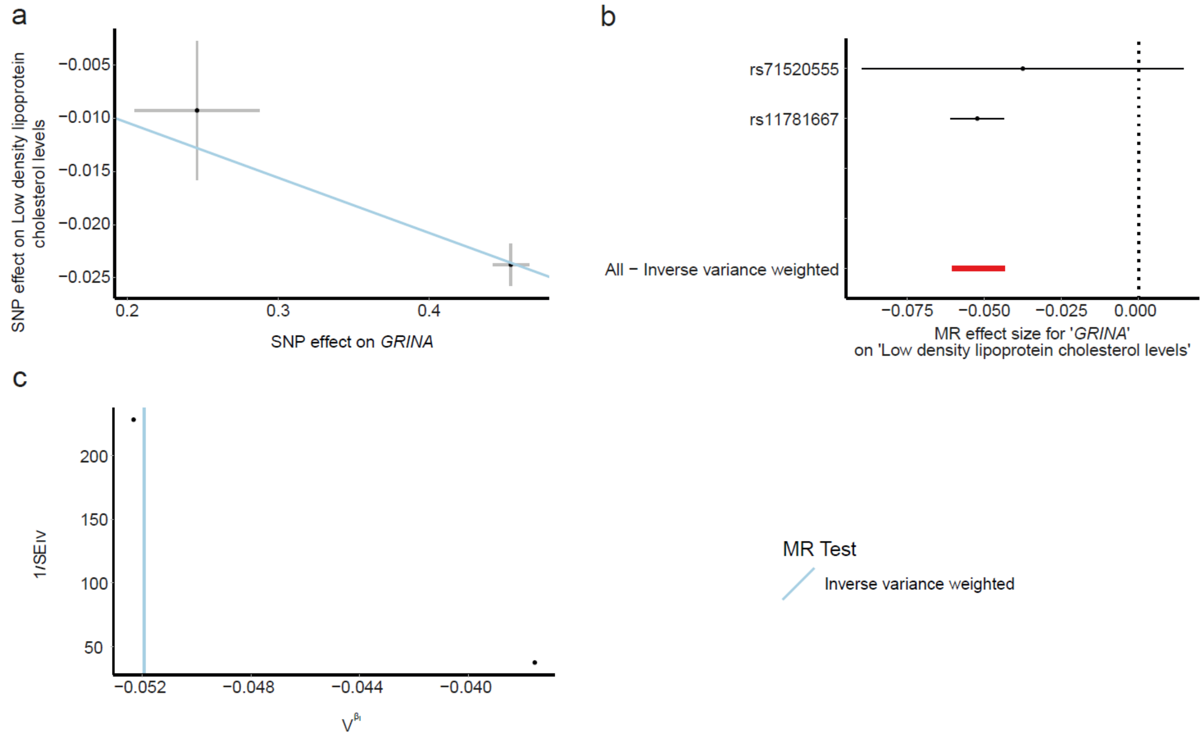

ESM Fig. 12 Plots MR base results *GRINA* on LDL-cholesterol. **A.** Scatterplot of SNP effect *GRINA*. **B.** Forest plot of MR effect size *GRINA* on LDL-cholesterol. **C.** Funnel plot for distribution of instruments.

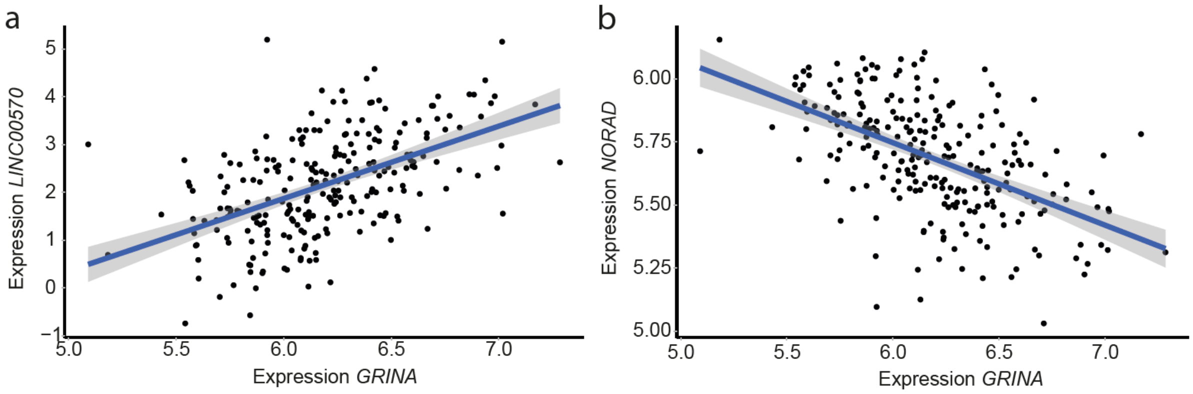

ESM Fig. 13 Scatterplot of expression *GRINA* versus *LINC00570* and *NORAD*.
A. Scatterplot of expression *GRINA* versus *LINC00570*. Spearman correlation coefficients 0.536. **B.** Scatterplot of expression *GRINA* versus *NORAD*. Spearman correlation coefficients -0.569.

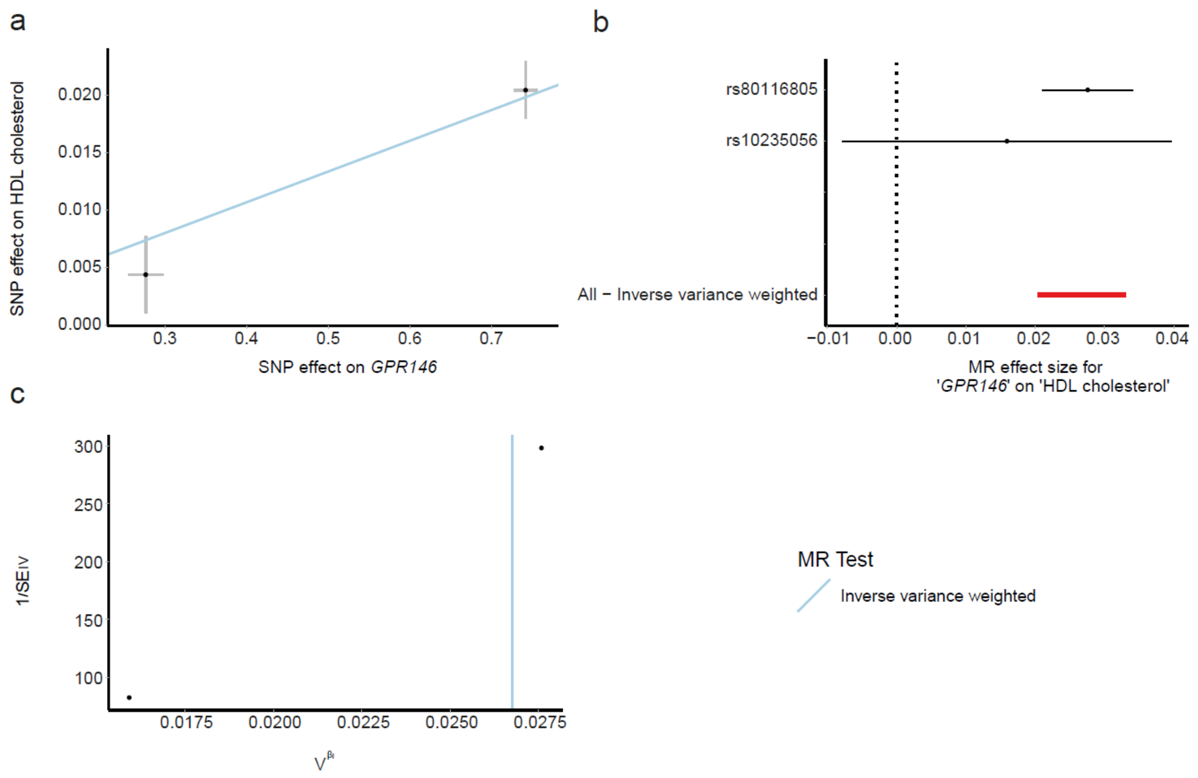

ESM Fig. 14 Plots MR base results *GPR146* on HDL-cholesterol. **A.** Scatterplot of SNP effect *GPR146*. **B.** Forest plot of MR effect size *GPR146* on HDL-cholesterol. **C.** Funnel plot for distribution of instruments.

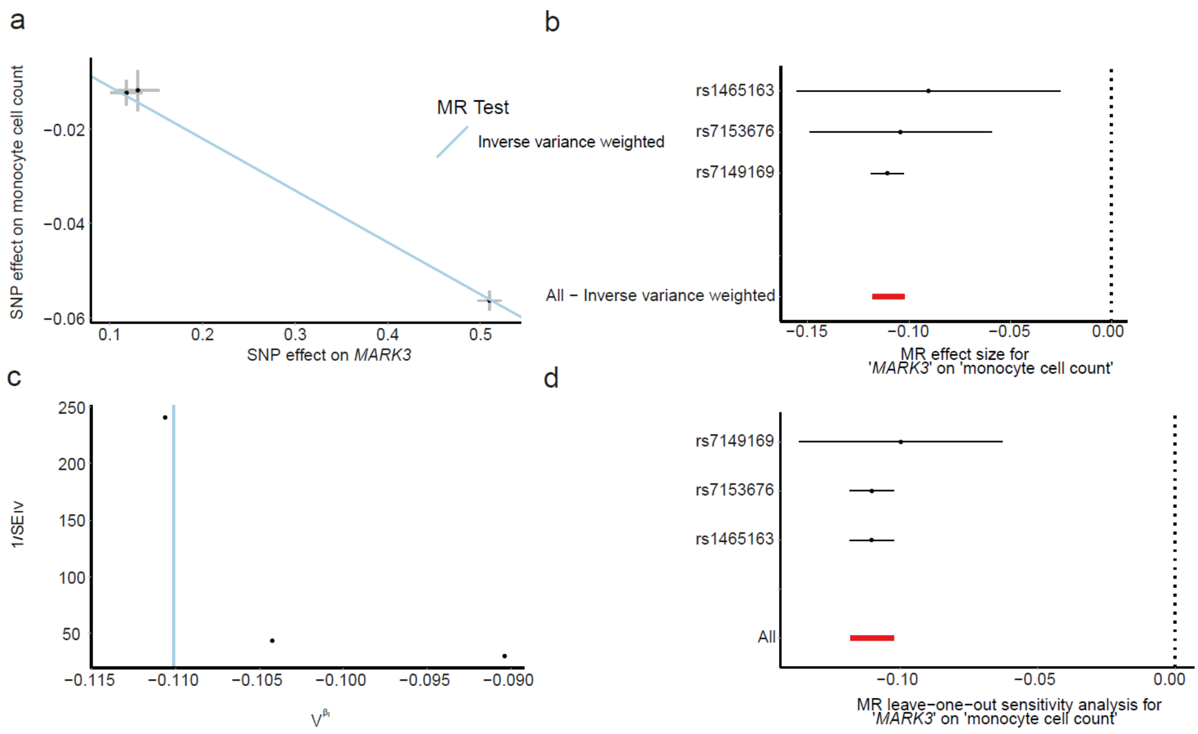

ESM Fig. 15. Scatter plot and Forest plot of MR base results *MARK3* on monocyte cell count. **A.** Scatterplot of SNP effect *MARK3*. **B.** Forest plot of MR effect size *MARK3* on monocyte cell count. **C.** Funnel plot for distribution of instruments. **D.** Leave one out plot.

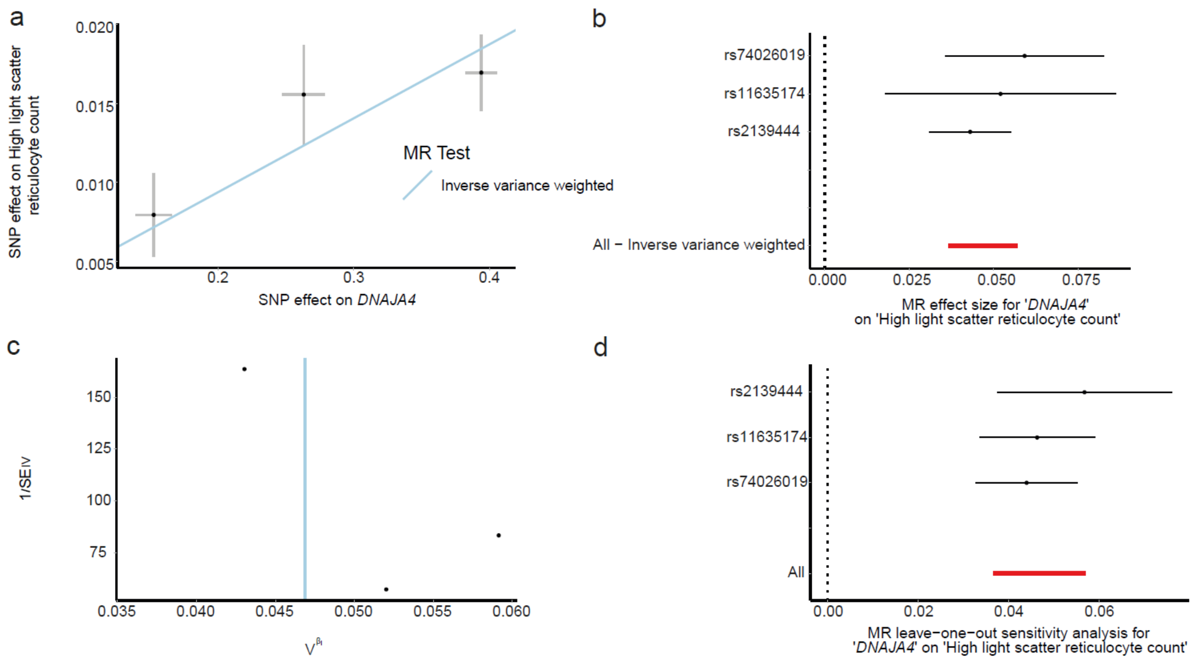

ESM Fig. 16 Scatter plot and Forest plot of MR base results *DNAJA4* on reticulocyte count. A. Scatterplot of SNP effect *DNAJA4*. **B.** Forest plot of MR effect size *DNAJA4* on reticulocyte count. **C.** Funnel plot for distribution of instruments. **D.** Leave one out plot.
